# Research on the Preparation of Supercapacitor Separators with High Wettability and Excellent Temperature Adaptability Through In Situ Deposition of Nano-Barium Sulfate on Regenerated Cellulose

**DOI:** 10.3390/polym17070842

**Published:** 2025-03-21

**Authors:** Hui Li, Jiehua Li, Chuanshan Zhao, Fenfen Zhao

**Affiliations:** 1 State Key Laboratory of Green Papermaking and Resource Recycling, Qilu University of Technology, Shandong Academy of Sciences, Jinan 250353, China; 15106900618@163.com (H.L.);; 2 Faculty of Arts, Shandong Shenghan Finance and Trade Vocational College, Jinan 250316, China

**Keywords:** separators, Lyocell fibers, fibrillation, nano-barium sulfate, physical properties, ionic properties, charge–discharge performance, temperature adaptability

## Abstract

In this paper, environmentally friendly separator materials with high mechanical and electrochemical properties were prepared from regenerated cellulose. This was achieved by studying the drawbacks of existing supercapacitor separators and then preparing protofibrillated fibers and nanofibrillated cellulose. The process involved the in situ deposition of nano-BaSO_4_ using paper milling and papermaking techniques. The separators were tested for a tensile strength of 47.25 MPa, puncture strength of 156 gf, and tear strength of 8.9 KPa-m^2^/g; uniform pore size (0.6–2 μm) and abundant porosity (81.3%); good wettability (9.2°) and water absorption; and excellent temperature resistance (no deformation at 180 °C), as well as good temperature adaptability from −40 °C to 100 °C. This simple process, suitable for mass production, enables the development of a new separator material with great application potential.

## 1. Introduction

The rapid development of portable electronic devices and new energy vehicles has placed significant demands on advanced energy storage systems, which have become one of the most critical materials in these fields. These devices are characterized by high charge storage capacity and power density [1,2,3]. Among various energy storage devices, supercapacitors are considered highly promising, with examples including graphene helium-ion batteries, fuel cells, and solid-state electrolytic capacitors. The separator plays a crucial role in the structure of supercapacitors, serving a dual function: acting as a physical barrier between the two electrodes while providing conductive pathways for efficient ion transport [4,5,6,7,8,9]. Consequently, the parameters of high-performance separators significantly enhance the overall performance of supercapacitors (SCs). An ideal separator should exhibit high mechanical strength to ensure structural integrity during operation, high porosity to facilitate efficient ion transport, good hydrophilicity to improve electrolyte wettability, excellent thermal stability to maintain functionality across varying temperature conditions, and stable chemical properties to resist degradation and ensure long-term reliability [10,11,12,13].

Nevertheless, due to the exacting demands placed on them, separators with comprehensive advanced properties have largely remained inadequately explored. To date, two principal categories of separators have found the most extensive application in commercial supercapacitors: polyolefins and nonwoven fabrics [14,15,16]. Polyolefin materials, such as polypropylene (PP) spacers, have a certain degree of durability. However, due to their relatively low glass transition and melting temperatures, they shrink at high temperatures. In addition, their porosity and hydrophilicity impede ion transport. In contrast, nonwoven supercapacitor separators, such as nonwoven polypropylene variants, have good porosity and hydrophilicity. However, the meltblown manufacturing process reduces their mechanical strength and reliability, which reduces safety and stability. To overcome these huge bottlenecks, a large number of researchers have devoted themselves to exploring advanced manufacturing methods and improved technologies for separators [17,18,19]. Nevertheless, the high-temperature performance of composite separators is not yet optimal due to the inherent limitations of polyolefin-based separators. Glass fiber separators, renowned for their high ionic conductivity, exemplary electrolyte uptake, and noteworthy thermal stability, are frequently utilized as separators. Nevertheless, their substantial cost and thickness present significant impediments to their large-scale industrial implementation [20,21,22]. In recent years, a variety of processes have been utilized to develop novel separator structures. These include electrostatic spinning [23,24,25], bio-stripping [26,27,28,29], environmentally friendly synthesis methods, and advanced drying technologies [30,31]. However, these processes are often complex, costly, and difficult to scale up. Consequently, the development of low-cost, simple, environmentally friendly, and large-scale production-ready separators with optimal overall performance remains a significant challenge. To overcome this challenge, it is essential to overcome the technological limitations through interdisciplinary innovation in materials science, manufacturing technology, and chemistry.

Cellulose, the most abundant biodegradable natural polymer material on Earth, is an ideal candidate for separator materials due to its low cost, good hydrophilicity, and excellent chemical stability [32,33]. The amalgamation of these properties is poised to address the salient limitations of extant separator technologies. Derivatives such as cellulose acetate, methyl cellulose, and microcrystalline cellulose have been prepared as battery separators, prepared through electrostatic spinning or phase conversion methods [34,35,36]. Regenerated cellulose separators have been shown to possess exceptional mechanical strength and thermal stability, a result of their robust hydrogen bonding and van der Waals forces. This enhances their capabilities, surpassing the limitations typically associated with cellulose-based separators. Research has demonstrated that regenerated cellulose separators possess considerable potential in the domain of energy storage, thereby propelling the advancement of high-performance cellulose separators. Notably, among the various types of regenerated cellulose fibers, Lyocell fiber, in particular, has been observed to be susceptible to protofibrillation under conditions of moisture or mechanical stress [37]. This phenomenon can be attributed to the high axial orientation of the Lyocell fibers, along with the relatively weak lateral bonding between the microfibers. This weakening of the bonding force is further exacerbated by fiber swelling in the wet state. Consequently, cortical fibers tend to detach, while the remaining cortical fibers undergo longitudinal cleavage and eventually form non-homogeneous fibers [38]. The fiber structure formed after fibrillation can intricately mesh with each other, which significantly improves the overall mechanical strength of the separator material and gives it better structural stability in similar battery applications. The material exhibits adjustable pore size, appropriate porosity, excellent wettability, and high thermal stability. Consequently, it is regarded as a promising candidate material for supercapacitor separator research, with the potential to enhance energy storage performance [39,40]. A comparison of fibrillated cellulose and bacterial cellulose with the original fibrillated fiber reveals notable distinctions. The original fibrillated fiber possesses a unique micro-nanometer size, making it particularly well-suited for the pulp and paper production process. Notably, its length-to-diameter ratio exceeds 300, a critical factor in achieving large-scale production. Furthermore, the fiber’s length-to-diameter ratio, which is more than 300, facilitates its utilization in the papermaking process, a crucial step in the production process. This characteristic confers a distinct advantage, namely a simplified process and reduced preparation cost, facilitating more expeditious realization of technological breakthroughs and, consequently, industrialized applications.

Extracted from natural plant resources such as wood and cotton, nanocellulose is renewable and biodegradable. Compared with traditional petroleum-based separation materials, nanocellulose is more environmentally friendly and more compatible with sustainable development [33,41,42]. Nanocellulose inherently has high strength and high modulus. Once integrated into the separator, it can interact with the separator’s matrix material via hydrogen bonds, van der Waals forces, and so on, creating a three-dimensional network structure. This interaction boosts the separator’s overall mechanical properties, like tensile strength and puncture resistance. As a result, the separator can more effectively preserve its structural integrity throughout the battery’s charge-discharge cycles. Meanwhile, nanocellulose can enhance the thermal stability of the material and regulate its pore structure [43,44,45,46,47].

Nano-barium sulfate is an inorganic material with a stable chemical structure, which has excellent structural enhancement properties and temperature and fire resistance. In the field of composite-coated separators, nano-barium sulfate as a surface coating can stabilize the separator in high-temperature environments, avoiding the contraction or melting of polyolefin-based separators at high temperatures and avoiding battery short circuits, which significantly improves the safety of the battery. In addition, its special particle morphology at the nanometer scale can improve the porosity of the separator, increase the wettability of the electrolyte, and improve the affinity of the electrolyte, thus promoting lithium-ion transport, lowering chemical impedance, and improving the electrochemical performance of the separator [48,49,50,51].

In this study, regenerated cellulose Lyocell fiber was used as the basic raw material. Primary fibrillated fibers with diameters ranging from 1 to 2 μm were successfully prepared through a wetting and swelling treatment followed by a fiber milling process. Subsequently, barium sulfate nanoparticles were generated in the nanocellulose matrix using an in situ deposition technique. They were then homogeneously mixed with the primary fibrillated fibers, and a composite regenerated cellulose separator was made using a papermaking process. This separator has a highly uniform porous NFRC-Ba structure, which not only helps to enhance its electrolyte wettability and facilitates efficient ion transfer but also plays an important role in structural enhancement and temperature resistance, ensuring durability under various operating conditions. The electrochemical performance of the separator was tested by subjecting it to high and low temperatures. The results showed excellent temperature adaptation from as cold as minus 40 degrees Celsius to a relatively high 100 degrees Celsius, which greatly expands the potential applications of the separator in extreme condition scenarios. In addition, it is most noteworthy that the preparation process of this composite regenerated cellulose separator is based on a papermaking process, which is simple, low-cost and can be produced on a large scale by using special industrial papermaking equipment, making this composite regenerated cellulose (NFRC-Ba) separator the ideal separator material for advanced supercapacitors, as well as for other high-performance, high-security capacitors on the market.

## 2. Materials and Methods

### 2.1. Materials and Reagents

Lyocell fibers (CLY), supplied by Shandong Boao New Material Co., Ltd. (Linqing, China), had an average length of 4 mm and a diameter of 1.33 denier. Nanocellulose (NFC), with a length ranging from 0.5 to 1 μm and a diameter of 50–100 nm, was NFRC-Ba, which was purchased from Shandong Shengquan Group (Jinan, China). Sodium sulfate (analytical reagent, AR) and barium chloride (analytical reagent, AR) were procured from Sinopharm Chemical Reagent Co., Ltd. (Shanghai, China).

### 2.2. Preparation of Composite Regenerated Cellulose Separator

**Figure 1:**
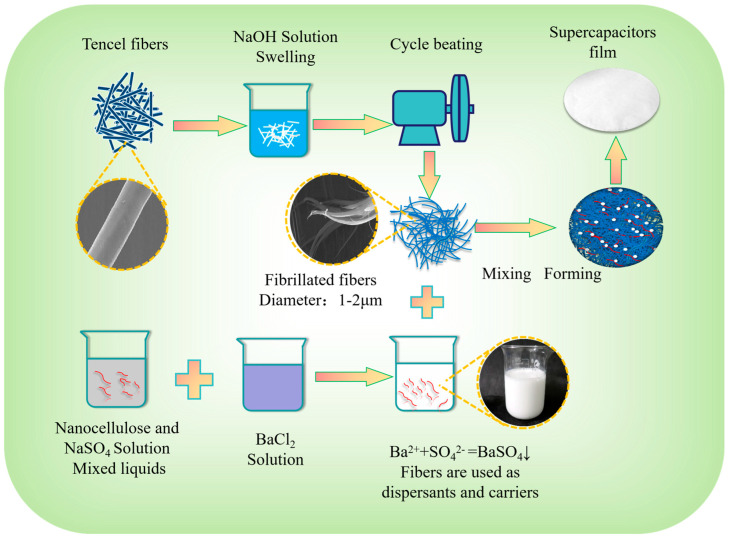
The preparation process of the composite regenerated cellulose separator paper.

1. In the initial step of the process, Lyocell fibers (CLY) were immersed in a 1 wt% sodium hydroxide solution for a duration of two hours. This step allowed the fibers to fully absorb the solution and undergo swelling, thereby weakening the hydrogen bonds between the fibers. Subsequently, the treated CLY was processed into a 10 wt% fiber stock and fed into a pulper (Shandong Dazhi Papermaking Equipment Co., Ltd., Zhuchengi, China) for fine grinding at an angular velocity of 80,000 rpm at a controlled grinding temperature of 60 °C. The resultant CLY fiber stock exhibited a consistency of 10 wt%. Through this process, the fibers underwent complete fibrillation, ultimately yielding primary fibrillated fibers (MCLY) with a diameter ranging from 1 to 2 μm.
2. A 1 M solution of sodium sulfate and a 1 M solution of barium chloride were prepared. The sodium sulfate solution was added to a 3 wt% nanofibrillar cellulose (NFC) mixture in a 5:5 ratio. Then, an ultrasonic disperser (Jining AoChao Electronic Equipment Co., Ltd., Jiningi, China) was used to disperse the NFC system for 30 min at 25 °C to ensure complete dispersion. Subsequently, the barium chloride solution was introduced into the NFC mixture in accordance with the molar ratio of sulfate ions (SO4^2−^) to barium ions (Ba^2+^) of 1:1, thereby inducing the precipitation of barium sulfate nanoparticles within the NFC system and the effective formulation of the NFC-BA mixture. This process occurs when barium ions in the solution react with sulphate ions in the presence of a regenerated cellulose matrix. Negatively charged functional groups on the cellulose surface attract positively charged barium ions and these adsorbed barium ions subsequently react with the sulphate ions in solution to form barium sulphate nanodeposits on the cellulose fibers.
3. MCLY and NFC-Ba were meticulously amalgamated in a precise proportion of 7:3, yielding a homogeneous liquid with a fiber concentration of 0.3 wt%. Composite regenerated cellulose separator paper (NFNFRC-Ba) with a basis weight of 13 g/m^2^ was fabricated using a dedicated paper machine. Concurrently, cellulose separator paper (FNFRC-Ba) with an identical basis weight (13 g/m^2^) to the control sample was fabricated solely utilizing MCLY as the raw material. The comprehensive preparation process for the composite regenerated cellulose separation paper (NFNFRC-Ba) is depicted in Figure 1.

## 3. Results

### 3.1. Physical Properties of the NFRC-Ba

**Figure 2:**
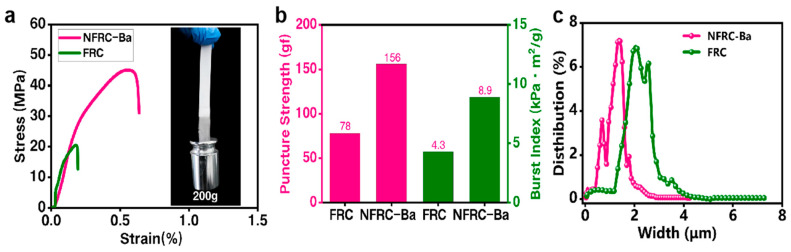
Physical strength and pore size distribution of NFRC-Ba and FRC separators: (**a**) stress-strain curve of NFRC-Ba/FRC separators; (**b**) bursting strength and puncture strength of NFRC-Ba/FRC separators; (**c**) pore size distribution of NFRC-Ba/FRC separators.

The mechanical strength of the separator is a critical parameter that has a profound effect on the safety of supercapacitors. An optimal separator should have sufficient mechanical strength to withstand potential damage during assembly and collisions during normal use [52]. Initially, the stress-strain properties of the NFRC-Ba and FRC separators were tested, as shown in Figure 2a. The overall fracture strength of the FRC was only 20.46 MPa. In contrast, the overall fracture strength of NFRC-Ba reached 47.25 MPa, which was 130.93% higher than that of the FRC separator. Furthermore, a tensile test using a 200 g weight verified the good dimensional stability and excellent mechanical strength of NFRC-Ba.

Concurrently, the NFRC-Ba separator exhibits noteworthy puncture and burst strengths, as illustrated in Figure 2b. The FRC separator demonstrates an overall puncture strength of 78 gf and a burst strength of 4.3 KPa·m^2^/g. In comparison, the NFRC-Ba separator exhibits a puncture strength of 156 gf and a burst strength of 8.9 KPa-m^2^/g, which is 2- and 2.1-fold greater than that of the FRC separator, respectively. These superior mechanical properties contribute to the preservation of the structural integrity of the separator in the event of an accidental impact, thereby averting rupture and significantly enhancing battery safety [53,54].

The underlying reasons for this disparity in mechanical strength can be elucidated as follows: MCLY fibers, being regenerated cellulose fibers, possess a smooth surface with a limited number of exposed hydroxyl groups. This characteristic leads to the presence of weak hydrogen bonding interactions between adjacent fibers, consequently resulting in inadequate mechanical strength of FRC separates made from MCLY fibers alone. In NFRC-Ba, the incorporation of NFC as a fiber-reinforcing additive yielded notable outcomes. In the domain of nanofibrillar cellulose research, NFC has been identified as an effective inter-fiber binder. By augmenting the density of hydroxyl groups, NFC enhances the hydrogen bonding between fibers, thereby enhancing the overall mechanical properties of the separator. The structural stability of nano-barium sulfate plays a significant role in the material enhancement process. The material exhibits high porosity, allowing for effective filling of the pores, thereby increasing the overall density and molecular bonding force. Consequently, this leads to an enhancement in the strength of the NFRC-Ba separator.

The uniform pore size and high porosity of the separator create a safe and stable channel for ion transport, which retains a large amount of electrolyte and enhances the ion-gap effect. This, in turn, improves the cycling performance and rate capability of supercapacitors. The pore diameters and porosities of the NFRC-Ba and FRC separators were measured using the piezomercury method. As illustrated in Figure 2c, the NFRC-Ba separator exhibits a comparatively narrow pore size distribution, predominantly concentrated within the range of 0.6 μm to 2 μm. The distribution curves for the NFRC-Ba separator demonstrate a distinct peak that approaches the median of the pore size distribution. In contrast, the distribution of the FRC separator exhibited a significantly wider range, extending from 1.3 μm to 3.5 μm, and its distribution curve exhibited two peaks, indicative of a comparatively poor uniformity of pore size distribution. The fabricated FRC separator demonstrated a porosity of approximately 56.7%, while the NFRC-Ba separator exhibited a porosity of approximately 81.3%. This value exceeds the porosity of the commercial NKK30AC-100 separator (53.4%) and the Celgard 2500 separator (38%). The NFRC-Ba separator features a uniform pore size and exceptional porosity, attributable to the reduced fiber diameter of protofibrillated fibers, which amplifies the specific surface area of the structure. The special irregular surface morphology of nano-barium sulfate, which fills the fiber pores, reduces the pore diameter of the separator and increases the overall surface area of the material. This results in a 24.6% increase in the porosity of NFRC-Ba compared with that of FRC. This significant contribution to the improvement of separator wettability and ion mobility is a notable finding. The incorporation of nanofibrillated cellulose into the composite further enhances its hydrophilicity, a consequence of the enrichment of hydrophilic groups (-OH) in the nanofibrillated cellulose. In summary, the uniform pore size and high porosity of the NFRC-Ba separator underscore its considerable promise for utilization in advanced supercapacitors and batteries.

**Figure 3:**
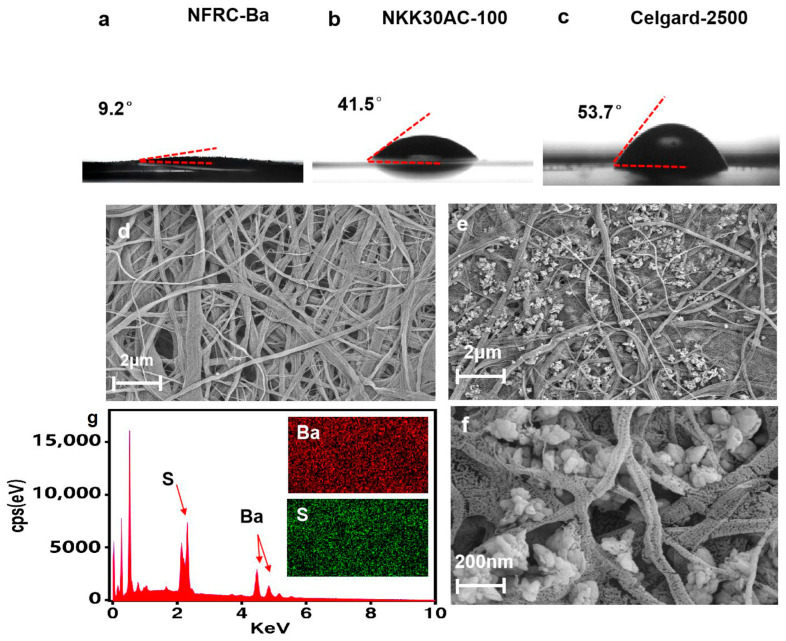
The electrolyte wettability tests of different separators: (**a**) NFRC-Ba separator; (**b**) NKK30AC-100 separator; (**c**) Celgard 2500 separator. SEM and EDS images of the surfaces of the NFRC-Ba and FRC separators: (**d**) SEM of FRC separators at low magnification; (**e**) SEM of NFRC-Ba separators at low magnification; (**f**) SEM of NFRC-Ba separators at higher magnification; (**g**) EDS distribution of S and Ba of NFRC-Ba separators.

The electrolyte wettability of the spacer is a critical parameter that has a profound effect on the electrochemical performance of supercapacitors and their assembly process [55]. The wetting characteristics of the different separators were evaluated by measuring the contact angle (CA). As shown in Figure 3a, the contact angle of a water droplet on the NFRC-Ba separator was initially measured to be 9.2°. More importantly, this value decreases to zero in only 5 s, in contrast to the NKK30AC-100 separator, which has a contact angle of 41.5°, and the Celgard 2500 separator, which has a contact angle of 53.7°, indicating that they are relatively less hydrophilic. This indicates that the NFRC-Ba separator is highly hydrophilic. Due to the hydrophilic nature of the micro- and nanostructured materials of the NFRC-Ba separators and the high porosity of the NFRC-Ba separators, the NFRC-Ba separators exhibit excellent electrolyte wetting. This property effectively accelerates electrolyte permeation, thereby increasing the ion transfer rate within the separator [56].

We examined them by scanning electron microscopy (SEM) (Figure 3d,e). Compared with FRC, the surface of NFRC-Ba separator was more compact and uniform. The nanofibrillated cellulose (NFC) fibers were bonded and reinforced on the surface of the protofibrillated fibers, filling the voids between the protofibrillated fibers. And the nano-BaSO_4_ was attached to the fiber surfaces and pores and uniformly distributed, which made the NFRC-Ba surface denser without unevenly sized pores like the FRC surface. Meanwhile, the structure of BaSO_4_ separator is denser, and the fiber pores are connected by CNF and nano-BaSO_4_, while the inter-fiber weave of FRC is looser, which also proves that the mechanical strength of NFRC-Ba is higher than that of FRC separator. The SEM images, magnified 30,000 times (Figure 3f), show the uniform nanoscale protofibrillated fiber morphology and BaSO_4_ surface structure, which makes the pore size and pore space of the separator more stable. Meanwhile, the pore size range is narrower, and a three-dimensional meander network is formed by multilayer interlacing, which increases the porosity and allows uniform migration of ions between electrodes. The energy dispersive spectra (EDS) of S and Ba elements in Figure 3g show the energy peaks of both elements, and the distribution of both elements in the material also verifies the uniform dispersion of nano-BaSO_4_ in the NFRC-Ba separator. The microstructural analysis demonstrates the excellent micro- and nanopore structure of the NFRC-Ba separator and also proves that the distribution and size of the pores can be optimized by precisely controlling the in situ deposition technique of nanocellulose and nano-BaSO_4_. The formation of curved ion channels increases the wettability and porosity of the separator, which can shorten the ion diffusion path, reduce the collision between ions, and improve the performance of supercapacitors (SCs) [57,58].

**Figure 4:**
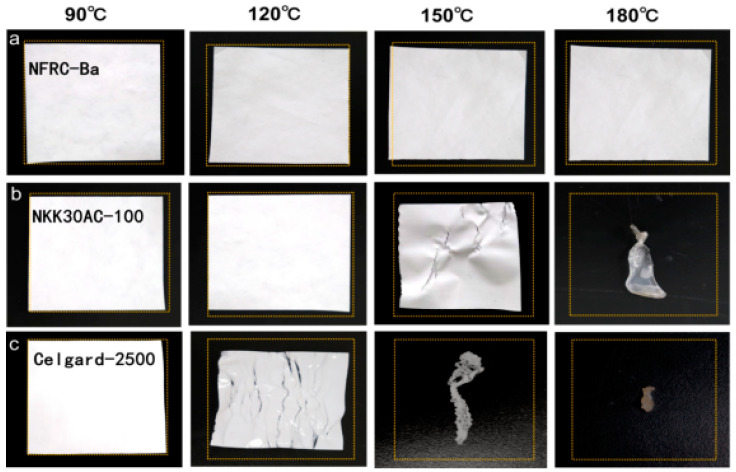
An illustration of the thermal shrinkages of the separators at different temperatures: (**a**) NFRC-Ba separator; (**b**) NKK30AC-100 separator; (**c**) Celgard 2500 separator.

In the area of ultracapacitor safety, the thermal stability of the separators is critical, especially in high-power and high-energy applications. During the charge/discharge cycle of an ultracapacitor, the heat released can damage the separator, resulting in potential short circuits or even explosions [59]. Figure 4a shows that the shrinkage of the NFRC-Ba separator is negligible at less than 3%, over a temperature range from ambient to 180 °C. The NFRC-Ba separator has been shown to retain its shape and dimensions even after repeated heat treatments. It effectively maintains its shape and size even after repeated heat treatments. On the other hand, the NKK30AC-100 separator is thermally stable up to 140 °C, but begins to wrinkle at 150 °C and melts at 180 °C (Figure 4b). The Celgard 2500 separator starts to thermally shrink at 120 °C and melts at 150 °C (Figure 4c). These results show that the NFRC-Ba separator has very high thermal stability due to its excellent raw material and fabrication technology. This is mainly due to the good high-temperature resistance of nano-BaSO_4_, which enhances the temperature adaptability of the separator, allowing the separator to maintain good dimensional stability at both high and low temperatures. This property is critical to mitigate safety concerns for supercapacitors and other batteries that operate at high temperatures.

A variety of interactions have been observed between barium sulfate nanoparticles and cellulose fibers. On one hand, the surface of barium sulfate nanoparticles may form hydrogen bonds with the hydroxyl groups on the surface of cellulose fibers. This intermolecular force strengthens the bonding between the two, thereby improving the overall stability of the diaphragm and contributing to the enhancement of the affinity of the electrolyte. From a microstructural perspective, the deposition of barium sulfate nanoparticles modifies the pore structure of cellulose fibers, thereby increasing their porosity. This change promotes more uniform distribution, which is beneficial for the storage and transmission of electrolyte, thereby enhancing electrolyte affinity. In terms of thermal stability, nano-barium sulfate itself possesses high thermal stability, with a melting point of 1600 °C. In the presence of nanoparticles, the interaction with cellulose fibers can limit the movement of cellulose molecular chains at high temperatures, thereby enhancing the stability of the diaphragm in high-temperature environments. Taken together, the above results suggest that the NFRC-Ba separator, which is characterized by its superior mechanical properties, uniform pore size, excellent porosity, highly efficient electrolyte wettability, and remarkable thermal stability, promises to emerge as a favorable candidate material for commercial supercapacitor separators.

### 3.2. Ionic Property of the NFRC-Ba Separator

**Figure 5:**
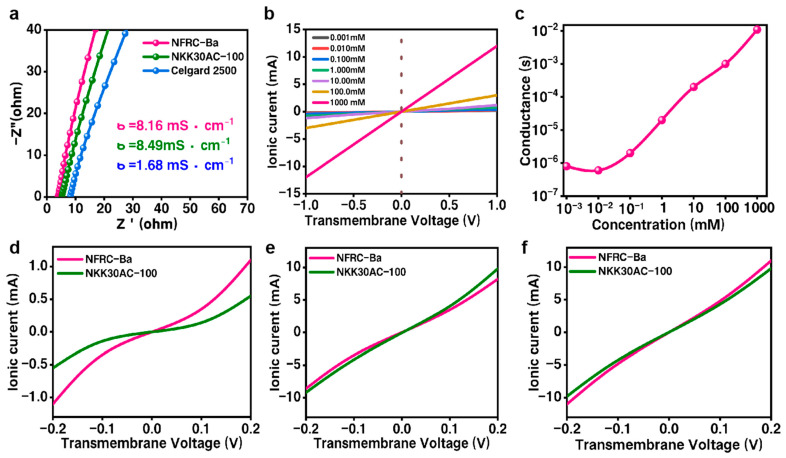
Ion Transport in Various Separators: (**a**) electrochemical impedance spectroscopy (EIS) of the NFRC-Ba, NKK30AC-100, and Celgard 2500 separators was utilized to calculate ionic conductivity; (**b**) the ionic current-voltage (I–V) behaviors of the NFRC-Ba separator were measured in KCl electrolytes with varying concentrations; (**c**) the trans-separator ion conductance of the separator was investigated as a function of electrolyte concentration. The I–V curves of the NFRC-Ba and NKK30AC-100 separators were obtained in commercial aqueous electrolytes; (**d**) 1.0 mol/L Na_2_SO_4_; (**e**) 1.0 mol/L H_2_SO_4_; and (**f**) 6.0 mol/L KOH.

The ionic conductivity of the NFRC-Ba separator, the Celgard 2500 separator, and the NKK30AC-100 separator in a sandwich structure was determined using electrochemical impedance spectroscopy (EIS). As demonstrated in Figure 5a, the EIS data indicate that the ionic conductivity of the NFRC-Ba separator is 8.16 mS/cm, a value that compares favorably with that of the commercial NKK30AC-100 separator (8.49 mS/cm) and greatly exceeds that of the Celgard 2500 separator (1.68 mS/cm). The excellent ionic conductivity of the NFRC-Ba separator is attributable to its wettability of the electrolyte, its absorption capacity and uniformity of pore size and high porosity [60]. Furthermore, an increase in ionic conductivity has been demonstrated to accelerate the rapid transport of ions in the separator, thus playing a key role in improving the electrochemical performance of the system.

A substantial body of research has demonstrated that the diffusion of ions in nanoporous materials can be substantially influenced by the localized electric field that is generated by the surface charge [61]. In view of this, a careful investigation was conducted into the ion transport behavior of the NFRC-Ba separator using a well-established ion current measurement technique. An aqueous KCl solution was selected as the electrolyte due to the similarity of the diffusion coefficients for K^+^ and Cl^−^ ions [62]. As demonstrated in Figure 5b, the ionic current-voltage (I–V) curves at varying concentrations manifest a distinct linear ohmic behavior, unmistakably indicating the occurrence of trans-separated ionic conductance. The ionic conductance of the bulk electrolyte was determined to be proportional to the concentration of the KCl electrolyte (Figure 5c). Specifically, at elevated concentrations, the ionic conductance conforms to the volume rule and displays a linear relationship. However, as the concentration gradually decreases, at 0.1 mM, the measured ionic conductance begins to deviate from the matrix value. In summary, the control of the surface charge on the ion transport in the trans-separator is minimal when the concentration of the electrolyte exceeds 0.1 mM. This finding serves to effectively confirm that surface charge exerts a negligible effect on the practical application of the NFRC-Ba separator.

**Figure 6:**
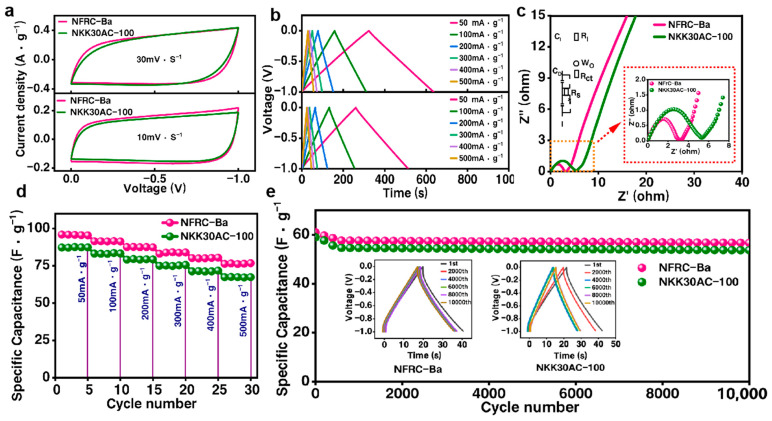
Comparison of Electrochemical Properties between the NFRC-Ba Separator and the Commercial NKK30AC-100 Separator in a 1.0 mol/L Na_2_SO_4_ Electrolyte: (**a**) cyclic voltammograms (CVs) of NFRC-Ba and NKK30AC-100 at different scan rates (10, 30 mV/s); (**b**) galvanostatic charge-discharge (GCD) curves of NFRC-Ba and NKK30AC-100 at various current densities ranging from 50 to 500 mA/g; (**c**) electrochemical impedance spectroscopy (EIS) curves of NFRC-Ba and NKK30AC-100; (**d**) specific capacitances of NFRC-Ba and NKK30AC-100 at different current densities; (**e**) long-term cycling performances of NFRC-Ba and NKK30AC-100 under a current density of 1 A/g.

The Debye–Hückel approximation suggests that, at elevated concentrations, the Debye screening length of the channels diminishes, approaching electrical neutrality. Conversely, at low concentrations, the electrical potential generated by surface charges leads to an augmentation of the Debye screening length. Furthermore, a specially designed cell was constructed in order to examine the ion transport capabilities of different separators in commercial aqueous electrolytes, namely 1.0 mol/L Na_2_SO_4_, 1.0 mol/L H_2_SO_4_, and 6.0 mol/L KOH. As demonstrated in Figure 5d,e, the current-voltage (I–V) curves illustrate that the NFRC-Ba separator displays a substantially higher ionic current in comparison to the NKK-MPF30AC-100 separator. The enhanced ion transport and permeability of the NFRC-Ba separator in the 1.0 mol/L Na_2_SO_4_ electrolyte, as illustrated in Figure 6, can be attributed to its high number of hydrophilic groups (-OH) and ion transport channels. These properties result in a 2.5-fold increase in ion transport and permeability compared to commercial reference separators. Conversely, in the 6.0 mol/L KOH electrolyte, the NFRC-Ba separator exhibits a reduced ionic current. This phenomenon can be attributed to the restriction of OH^−^ ion transport imposed by the negative surface charge.

### 3.3. Charge–Discharge Performance of the NFRC-Ba Separator

To assess the potential commercial viability of the NFRC-Ba separator, the researchers assembled symmetrical supercapacitors using the NFRC-Ba separator and a commercial NKK30AC-100 separator and measured their electrochemical properties separately using an electrochemical workstation (Shanghai Chenhua Instrument Co., Ltd., Shanghai, China). The electrochemical properties of the fabricated supercapacitors were evaluated using cyclic voltammetry (CV), galvanostatic charge/discharge (GCD), and electrochemical impedance spectroscopy (EIS) measurements. It was found that the electrodes and electrolytes were uniform for all devices, and that any observed differences in cell performance could therefore be attributed to the unique features of the separator.

As illustrated in Figure 6a, the cyclic voltammetry (CV) curves obtained for the fabricated supercapacitor immersed in a 1.0 M sodium sulfate electrolyte at 10 mV/s and 30 mV/s scan rates are presented. The rate performance of the NFRC-Ba separator is shown to improve with increasing scan rate, as evidenced by its nearly rectangular CV curve profile. Conversely, the CV curve of the NKK30AC-100 separator exhibits substantial distortion, indicative of its comparatively inferior rate performance, which may be ascribed to the elevated equivalent series resistance (ESR) within the device. Conversely, the CV curve of the NFRC-Ba separator is quasi-rectangular, indicating a fast current response during voltage reversal, thus suggesting that the equivalent series resistance of the device is low. Furthermore, the CV curve of the NFRC-Ba separator encompasses a substantially larger area than that of the NKK30AC-100 separator. This discrepancy in enclosed area is indicative of the NFRC-Ba separator possessing a higher specific capacitance. At a scan rate of 10 mV/s, the specific capacitance of the NFRC-Ba separator is approximately 32.45 F/g, which exceeds that of the NKK30AC-100 separator at 26.72 F/g. Furthermore, at an augmented scan rate of 30 mV/s, the capacitance retention of the NFRC-Ba separator attains 81.25%, which is considerably higher than that of the NKK30AC-100 separator at 72.98%.

Electrostatic charge/discharge testing is a common and accurate method for determining capacitance. In the majority of applications where supercapacitors are typically subjected to external loads, the operational behavior of electrostatic charging and discharging is more closely related to their electrochemical behavior [63]. Typical electrostatic charge/discharge curves of the fabricated supercapacitor collected over a current density range of 50–500 mA/g are shown in Figure 6b. The electrostatic charge/discharge curves of the NFRC-Ba separator exhibit excellent symmetry, suggesting optimal electrochemical performance. Concurrently, the discharge curve of the NFRC-Ba separator demonstrates a more pronounced IR drop, signifying a satisfactory equivalent series resistance. For instance, at a current density of 50 mA/g, the total charge/discharge time for the NFRC-Ba separator is 638 s, whereas for the NKK30AC-100, it is only 520 s. Furthermore, the NFRC-Ba separator exhibits superior performance in terms of charge/discharge time when compared to the NKK30AC-100 at various current densities, thereby substantiating its more uniform and stable performance.

Typical electrostatic charge/discharge curves of the fabricated supercapacitors collected in the current density range of 50–500 mA/g are shown in Figure 6c. The electrostatic charge/discharge curves of the NFRC-Ba separator have excellent symmetry, indicating the best electrochemical performance. At the same time, the discharge curve of the NFRC-Ba separator shows a more pronounced IR drop, indicating a satisfactory equivalent series resistance. For example, at a current density of 50 mA/g, the total charge/discharge time of the NFRC-Ba separator is 638 s, compared with 520 s for the NKK30AC-100. In addition, the NFRC-Ba separator shows superior performance in charge/discharge times at all current densities compared to the NKK30AC-100, demonstrating more uniform and stable performance.

Calculated from typical electrostatic charge-discharge curves, Figure 6d shows the single-electrode specific capacitance of the NFRC-Ba separator and the NKK30AC-100 separator at different current densities. As the current density increases from 50 mA/g to 500 mA/g, the specific capacitances of the NFRC-Ba separator reach 89.52 F/g, 87.36 F/g, 85.15 F/g, 82.25 F/g, 80.57 F/g, and 78.82 F/g, respectively, which are higher than those of the NKK30AC-100 separator at the same current density, i.e., 85.28 F/g, 81.36 F/g, 81.36 F/g, and 78.82 F/g, respectively. It is clear that the capacitance of the NFRC-Ba separator is significantly higher than that of the NKK30AC-100 separator. This is mainly due to its better electrolyte wettability and larger electrolyte ion transfer space. When the current density is increased to 500 mA/g, the capacitance retention of the SC-NFRC-Ba separator is about 87.48%, which is significantly higher than that of the NKK30AC-100 separator.

In addition, cycle life is one of the most important characteristics of high-performance separators [64]. Figure 6e shows the results of 10,000 cycles in a 1.0 M sodium sulfate electrolyte at a current density of 1 A/g. The capacitance of the NFRC-Ba separator remains almost unchanged, and its specific capacitance efficiency remains above 95%, while the specific capacitance efficiency of the NKK30AC-100 decreases to 91%, demonstrating the excellent cycling durability of the NFRC-Ba separator. This demonstrates the excellent cycling durability of the NFRC-Ba separator. As shown in the inset of Figure 6e, the long-term cycling test of the NFRC-Ba separator shows a relatively large IR drop. In addition, the discharge curve of the NKK30AC-100 separator shows a worse IR drop compared to the NFRC-Ba separator. In addition, when the supercapacitor was disassembled after 10,000 cycles, it was observed that the experimental NFRC-Ba separator still retained its original morphology. This is due to its special three-dimensional pore structure, with protofibrillated fiber as the skeleton and NFC and nano-BaSO_4_ as structural reinforcement and filling, providing good structural strength and durability.

In order to explore the performance of the NFRC-Ba separator in extreme environments, the electrochemical adaptation of the fabricated NFRC-Ba separator was investigated at temperatures ranging from high to low. Specifically, the electrochemical behavior of the NFRC-Ba separator was investigated in two temperature ranges: 25 °C to 100 °C and room temperature (RT) to −40 °C.

**Figure 7:**
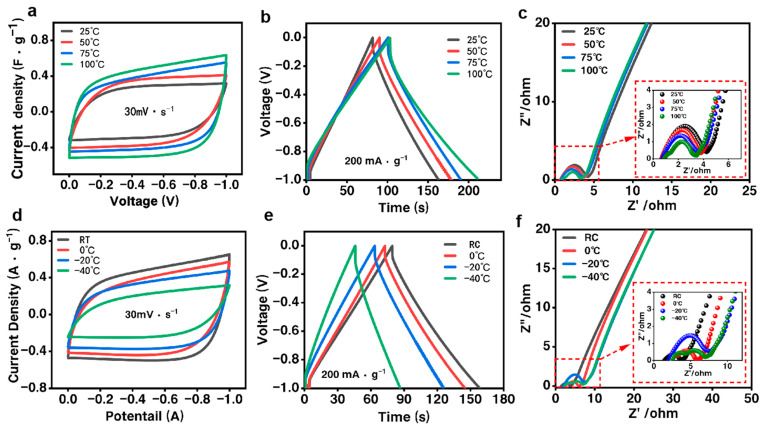
Comparison of Electrochemical Properties between the NFRC-Ba Separator and the Commercial NKK30AC-100 Separator at 25–100 °C: (**a**) cyclic voltammograms (CVs) of NFRC-Ba at a scan rate of 30 mV/s; (**b**) galvanostatic charge-discharge (GCD) curves of NFRC-Ba at a current density of 500 mA/g; (**c**) electrochemical impedance spectroscopy (EIS) curves of NFRC-Ba. Comparison of Electrochemical Properties between the NFRC-Ba Separator and the Commercial NKK30AC-100 Separator at RC to −40 °C: (**d**) cyclic voltammograms (CVs) of NFRC-Ba at a scan rate of 30 mV/s; (**e**) galvanostatic charge-discharge (GCD) curves of NFRC-Ba at a current density of 500 mA/g; (**f**) electrochemical impedance spectroscopy (EIS) curves of NFRC-Ba.

The electrochemical behavior of the NFRC-Ba separator was also investigated in two temperature intervals. As shown in Figure 7a–c, high-temperature application tests from 25 °C to 100 °C, and cyclic voltammetry (CV) curve tests in Figure 7a, indicate that increasing temperature enlarges the area enclosed by the CV curve, while its rectangular shape remains unchanged. This indicates that the NFRC-Ba separator has good ionic conductivity at high temperatures. The high-temperature electrostatic charge-discharge curve measured at a current density of 30 mA/g (shown in Figure 7b) shows a quasi-triangular curve similar to that at room temperature. It should be noted that the higher the temperature, the longer the charging and discharging time. The specific capacitances measured at 25 °C, 50 °C, 75 °C, and 100 °C were 63.29 F/g, 75.84 F/g, 79.23 F/g, and 82.38 F/g, respectively, showing remarkable capacitive performance. Figure 7c shows the electrochemical impedance spectroscopy (EIS) curves of the NFRC-Ba separator over the temperature range of 25 °C to 100 °C. The EIS curves are shown in Figure 7c. The decrease in interfacial resistance with increasing temperature is limited, ranging from 6 Ω at 25 °C to 3 Ω at 100 °C. In contrast, the decrease in series resistance is even greater. As the temperature increases from 25 °C to 100 °C, the series resistance decreases from 12 Ω to 8 Ω, mainly due to the increased ionic conductivity of the NFRC-Ba separator at high temperatures.

Separator and electrochemical adaptability are also particularly important at low temperatures, as shown in Figure 7d, where the area enclosed by the cyclic voltammetry (CV) curve shrinks as the temperature decreases from RC to −40 °C. Also, its rectangular shape starts to be irregular, which proves that the efficiency of the NFRC-Ba separator decreases at very cold temperatures despite working properly. The electrostatic charge/discharge curve measured at a current density of 30 mA/g at low temperatures (Figure 7e) shows a quasi-triangular shape similar to that at RC. In addition, the charge/discharge duration decreases with decreasing temperature. This indicates that the ionic conductivity of the NFRC-Ba separator is suppressed at low temperatures. The specific capacitances measured at RC, 0 °C, −20 °C, and −40 °C were 62.2 F/g, 53.4 F/g, 49.8 F/g, and 41.7 F/g, respectively, and 86%, 80%, and 67% of the capacitance was retained at 0 °C, −20 °C, and −40 °C, respectively, compared with that at RC. Figure 7f shows the electrochemical impedance spectral curve of the NFRC-Ba separator over the temperature range from RC to −40 °C. The curve is shown in Figure 7f. It is interesting to note that in the low-frequency region, the curve remains nearly vertical at almost all temperatures in this temperature range, reflecting the stable charge transfer capability of the NFRC-Ba separator. This stability can be attributed to the excellent adaptation of the separator to the low-temperature environment. The diffusion resistance of the supercapacitor increases with decreasing temperature due to slower ion transport at low temperatures. The increase in interfacial contact resistance with decreasing temperature is limited, ranging from 3 Ω at RC to 7 Ω at −40 °C. In contrast, the increase in series resistance is much greater. As the temperature decreases from RC to −40 °C, the series resistance increases from 6 Ω to 18 Ω, primarily due to the reduced ionic conductivity of the NFRC-Ba separator at low temperatures.

An experimental investigation was conducted to assess the performance indices of NFRC-Ba separators and to compare them with those of NKK30AC-100 and Celgard-2500 separators. The experimental findings demonstrated that NFRC-Ba separators exhibited superior pore size, porosity, thermal stability, and chemical stability. As delineated in Table 1, the findings reveal a comparison of various parameters. With regard to mechanical strength, the NFRC-Ba separator exhibits a lower value compared to the NKK30AC-100 separator and Celgard-2500 separator. This is attributable to the composition of the two types of separators, which are made of plastic material membranes. Consequently, they possess enhanced mechanical strength. However, their plastic products exhibit significant defects in wettability, porosity, and thermal stability. The electrochemical performance of the separator can be determined by measuring several indicators. These indicators are pivotal in determining the electrochemical performance of the separator. The data presented in the table demonstrate a clear correlation between enhanced wettability, porosity, and thermal stability parameters and improved electrochemical performance. Furthermore, with respect to extreme temperature adaptability, the NFRC-Ba separator demonstrates a significant advantage over the NKK30AC-100 and Celgard-2500 separators. This finding underscores the efficacy of the high-performance supercapacitor separator developed and evaluated in this study, highlighting its significant potential in the realm of advanced and high-performance supercapacitors.

## 4. Discussion

In this paper, Saiwen fiber was used as the raw material. The fiber modification treatment, including the wet milling process to prepare micro-nanometer grade protofibrillated fibers, was followed by nanofibrillated cellulose as the structural reinforcement and binder. Barium sulfate nanoparticles were then deposited in situ on the surface of the fibers. Finally, a high-performance supercapacitor was prepared using papermaking fiber molding technology. This method not only uses cheap raw materials but also simplifies the preparation process by utilizing existing papermaking techniques to fully realize industrial production. In addition, we conducted several performance tests on the prepared separator, including tests of its physical properties (mechanical strength, porosity, hydrophilicity, thermal stability), ionic properties (ionic conductivity, ionic transport ability), and charge/discharge performance (specific capacitance, capacitance retention, cycle life, and temperature extremes), and compared them with the commercial separator. The prepared separator demonstrated 47.25 MPa in tensile strength, 156 gf in puncture strength, and 8.9 KPa-m^2^/g in fracture strength; uniform pore size (0.6 μm–2 μm) and abundant porosity (81.3%); good wettability (9.2°) and absorbency; and excellent temperature resistance (no deformation at 180 °C). It also has a uniform and dense three-dimensional spatial structure and ion channels, which enhances its electrochemical performance. The ionic conductivity was measured at 8.16 mS/cm, and the specific capacitance reached 32.45 F/g, with a specific capacitance efficiency of more than 95% after 10,000 cycles. In addition, it passed extreme temperature performance tests, proving that the separator can function from −40 °C to 100 °C. In summary, NFRC-Ba separators have great potential as commercial materials for high-performance, high-reliability supercapacitors and other capacitors due to their excellent properties and extremely simple preparation process.

## Figures and Tables

**Table 1:** Data comparison of NFRC-Ba separators with commercial diaphragms.

Performances		NFRC-Ba Separator	NKK30AC-100 Separator	Celgard-2500 Separator
Mechanical Strength	Tensile Strength	47.25 MPa	100	117.68 MPa
	Puncture Strength	156 gf	300 gf	300 gf
	Fracture Strength	8.9 KPa-m^2^/g	12 KPa-m^2^/g	15 KPa-m^2^/g
Hole Diameter		0.6–2 μm	0.02–0.1 μm	0.02–0.5 μm
Porosity		81.3%	55%	55%
Contact Angle		9.2°	41.5°	53.7°
Thermal Stability		>180 °C	<140 °C	<110 °C
Electrochemical Properties ^1^	Ionic Conductivity	8.16 mS/cm	8.49 mS/cm	1.68 mS/cm
	Specific Capacitance	32.45 F/g	26.72 F/g	23.54 F/g
	Cyclical Durability	>95%	<95%	<95%
	Temperature Adaptability	−40–100 °C	RC	RC

^1^ The electrochemical performance is affected by factors such as the electrolyte of the manufactured super but container electrodes. The measured data are used for control comparisons only.

## Data Availability

The original contributions presented in this study are included in the article. Further inquiries can be directed to the corresponding authors.
